# MicroRNAs of Human Herpesvirus 6A and 6B in Serum and Cerebrospinal Fluid of Multiple Sclerosis Patients

**DOI:** 10.3389/fimmu.2020.02142

**Published:** 2020-09-18

**Authors:** María I. Domínguez-Mozo, Alejandro Nieto-Guerrero, Silvia Pérez-Pérez, María Á. García-Martínez, Rafael Arroyo, Roberto Álvarez-Lafuente

**Affiliations:** ^1^Environmental Factors in Degenerative Diseases Research Group, Instituto Investigación Sanitaria San Carlos (IdISSC), Hospital Clínico San Carlos, Madrid, Spain; ^2^Neurology Department, Hospital Universitario Quirónsalud Madrid, Madrid, Spain

**Keywords:** multiple sclerosis, human herpesvirus 6A/B (HHV-6A/B), microRNA, anti-HHV-6A/B IgG, intrathecal HHV-6A/B antibody production, anti-HHV-6/B IgM

## Abstract

Human herpesvirus-6A (HHV-6A) and −6B (HHV-6B) might be involved in the etiopathogenesis of multiple sclerosis (MS), especially the HHV-6A. We aim at assessing, for the first time in the scientific literature, the HHV-6A/B microRNAs in MS patients. We analyzed the miRNAs of HHV-6A: miR-U86, and −6B: hhv6b-miR-Ro6-1, −2, −3-3p, −3-5p, and −4 in paired samples of serum and CSF of 42 untreated MS patients and 23 patients with other neurological diseases (OND), using Taqman MicroRNA Assays. Intrathecal HHV-6A/B antibody production and anti-HHV-6A/B IgG/IgM levels in serum were measured. MS clinical data were available. We detected the following miRNAs: hhv6b-miR-Ro6-2 (serum: MS:97.7%, OND:95.7%; CSF: MS:81%, OND:86.4%), 3-3p (serum: MS:4.8%, OND:0%; CSF: MS:2.4%, OND:4.5%), −3-5p (serum: MS:95.2%, OND:91.3%; CSF: MS:50%, OND:54.5%), and miR-U86 (serum: MS:54.8%, OND:47.8%; CSF: MS:11.9%, OND:9.1%). In the serum of the whole population (MS and OND patients) we found a significant correlation between the levels of hhv6b-miR-Ro6-2 and −3-5p (Spearman *r* = 0.839, pcorr = 3E-13), −2 and miR-U86 (Spearman *r* = 0.578, pcorr = 0.001) and −3-5p and miR-U86 (Spearman *r* = 0.698, pcorr = 1.34E-5); also in the CSF, between hhv6b-miR-Ro6-2 and −3-5p (Spearman *r* = 0.626, pcorr = 8.52E-4). These correlations remained statistically significant when both populations were considered separately. The anti-HHV-6A/B IgG levels in CSF and the intrathecal antibody production in positive MS patients for hhv6b-miR-Ro6-3-5p were statistically significant higher than in the negative ones (pcorr = 0.006 and pcorr = 0.036). The prevalence of miR-U86 (30.8%) in the CSF of individuals without gadolinium-enhancing lesions was higher (*p* = 0.035) than in the ones with these lesions (0%); however, the difference did not withstand Bonferroni correction (pcorr = 0.105). We propose a role of HHV-6A/B miRNAs in the maintenance of the viral latency state. Further investigations are warranted to validate these results and clarify the function of these viral miRNAs.

## Introduction

Multiple sclerosis (MS) is a demyelinating inflammatory chronic disease that affects the central nervous system (CNS), and whose origin is probably autoimmune (1). After suffering the first episode of neurologic symptoms, referred to as clinically isolated syndrome (CIS), approximately 85 percent of patients with MS are initially diagnose with RRMS (relapse-remitting MS) (2). Although the etiology of MS is unknown, it is commonly accepted that an infectious agent could trigger the autoimmune reaction in genetically predisposed subjects (3). An extent number of published studies proposed the enrollment of the *Herpesviridae* family in this pathology, mainly the Epstein-Bar Virus (EBV) (4) and the human herpesvirus 6 (HHV-6A/B) (5).

HHV-6A/B is a ubiquitous human virus that belongs to the subfamily *Betaherpesviridae*. Its genome is a linear double-stranded DNA molecule of 159–162 kb consisting of a long unique (U) region flanked by terminal direct repeats (left DRL and right DRR) of 8–9 kb. Based on their temporal expression and their dependency on other gene products, its viral genes are divided into immediate-early (IE) genes, early (E) genes, and late (L) genes, like all herpesvirus (6). First, IE genes are expressed independently of *de novo* protein synthesis; their products are important regulators of E gene transcription, which are involved in DNA replication. Finally, L genes are transcribed, encoding structural and other proteins involved in virion assembly (7). Recently, HHV-6A/B has been classified as two distinct viruses, HHV-6A and HHV-6B. They share an overall identity of 90% out of their genome, but they differ in regards to their epidemiological, immunological, and biological properties, also to their disease association (8). Both HHV-6A and −6B are neurotropic, but the one classically associated with MS is the HHV-6A (9, 10). However, this association remains controversial, since most of the studies did not distinguish between HHV-6A and −6B (11, 12).

Because of the necessity of identified biomarkers in MS to help the diagnosis and stage of the disease, several studies are focusing on the study of microRNAs (miRNAs) in these patients (13, 14). However, not only the human cell synthesize miRNAs; at present, it has been described at least 100 miRNAs encoded by the human *Herpesviridae* family (15). Nevertheless, HHV-6B microRNAs were not identified until 2012 (hhv6b-miR-Ro6-1, −2, −3, and −4) (16) and the one from HHV-6A in 2015 (miR-U86) (17). HHV-6B miRNAs are expressed from both DRL and DRR, and in antisense orientation relative to IE open reading frame (ORF) (B1, B2, B2, and DR3) that has no homolog with HHV-6A. miR-U86 targets the HHV-6A IE gene U86.

Although the role of the other viral miRNAs from viruses likely involved in MS has already been investigated, such as the ones of the EBV (18), there is a lack of studies *in vivo* about the HHV-6A/B miRNAs. In this exploratory study, we aim at detecting the HHV-6A/B miRNAs in pairs of serum and cerebrospinal fluid (CSF) samples of a cohort of MS patients and look for a possible association with clinical variables related to the progression and activity of MS.

## Materials and Methods

### Patients and Samples

This retrospective observational case-control study included 42 MS patients and 23 patients with other neurological diseases (OND) from the Hospital Clínico San Carlos de Madrid, who were matched by age (MS: 38.3 ± 11.7, OND: 39.2 ± 13.1 years old) and gender (percentage of females: MS: 69%, OND: 65.2%) (Table 1). Among the MS patients, 23 had CIS at the recruitment (which developed into clinically definite MS later), and 19 had relapse-remitting (RR) MS according to the revised Mc-Donald criteria (19). All MS patients were not on disease-modifying treatments (DMTs). The subjects with OND comprised patients with: bilateral optic neuropathy (*n* = 1), conversion disorder (*n* = 4), headache (*n* = 3), ictus (*n* = 3), intracranial benign hypertension (*n* = 1), myelopathy (*n* = 2), neurosarcoidosis (*n* = 1), ophthalmoparesis (*n* = 1), paraparesis (*n* = 4), paresis sixth cranial nerve (*n* = 2) and pyramidal syndrome (*n* = 1); the inclusion criteria in the OND group was: patients with diseases not associated with HHV-6A/B infection. All the participants signed the informed consent. The local Ethics Committee of the Hospital Clínico San Carlos approved this study.

**Table 1:** Demographic and clinical data.

		**MS patients**			**OND patients**
		**All MS *n* = 42**	**CIS *n* = 23**	**RRMS *n* = 19**	***n* = 23**
Gender, males:females, (*n*:*n*)		13:29	8:15	5:14	8:15
Age at sampling, years (mean ± SD)		38.3 ± 11.7 *n* = 42	37.7 ± 12.6	39.1 ± 10.7	39.2 ± 13.1
MS disease duration, months [median (P25, P75)]		7 (1.8, 28) *n* = 42	2 (1, 8)	17 (7, 79)	
EDSS at sample collection [median (P25, P75)]		1 (0, 2) *n* = 38	1 (1, 0.5)	1.5 (0.8, 2.1)	
MSSS prior to treatment [median (P25, P75)]		2.7 (0.7, 5.2) *n* = 22	1.6 (0.6, 4.8)	3 (0.6, 5.2)	
Number of relapses at sample collection [median (P25,P75)]		1 (1, 2) *n* = 42	1 (1, 1)	2 (2, 3)	
Annual rate of relapses prior to treatment [median (P25,P75)]		1.2 (0.4, 3.3) *n* = 42	1.1 (0.4, 4.4)	1.2 (0.3, 2.5)	
Period of time: last relapse - sample collection, months [median (P25,P75)]		2.5 (0.75, 6) *n* = 42	2 (1, 8)	3 (0, 5)	
Radiological measurements	Number of gadolinium enhancing lesions [median (P25,P75)]	1 (0, 3) *n* = 28	0 (0, 2)	1 (0, 3)	
	Percentage of patients with more than 10 T2 lesions (%)	83 *n* = 41	91.3	72.2	

*CIS, clinically isolated syndrome; EDSS, Expanded Disability Status Scale; MS, multiple sclerosis; MSSS, Multiple Sclerosis Severity Scale; n, number of subjects; OND, other neurological diseases; P25, 25th percentile, P75, 75th percentile; RRMS, relapsing-remitting MS; SD, standard deviation*.

Paired samples of CSF and serum, collected from February 2010 until January 2015 and stored at −80°C on the same day of the extraction, were available for all patients. CSF was obtained by lumbar puncture performed by a diagnose purpose. Serum was obtained by vein puncture and isolated by centrifugation (920 g, 15 min, room temperature) in serum separator tubes.

### Analysis of HHV-6A/B miRNAs

Cell-free total RNA was extracted from serum or CSF using the miRNeasy Serum/Plasma Advanced Kit (Qiagen, Hilden, Germany) according to the manufacture's protocol. As suggested in this protocol, we added *C. elegans* miR-39 mimic and the bacteriophage MS2 RNA during the extraction process.

For reverse transcription (RT) and miRNA detection, specific primers from TaqMan miRNA assays (Thermo Fisher Scientific) were used. The analyzed miRNAs were: (1) HHV-6A/B miRNAs: hhv6b-miR-Ro6-1-5p (Taqman assay ID: 476063_mat), hhv6b-miR-Ro6-2-3p (473838_mat), hhv6b-miR-Ro6-3-3p (471439_mat), hhv6b-miR-Ro6-3-5p (473578_mat), hhv6b-miR-Ro6-4-3p (472738_mat), and miR-U86 (custom assay); (2) exogenous miRNA control: cel-mir-39-3p (478293_mir); and (3) endogenous miRNA control: hsa-miR-126-3p (002228), previously used in MS study (20).

The RT was carried out following the manual instruction of the TaqMan^®^ MicroRNA Reverse Transcription Kit (Thermo Fisher Scientific) in a Veriti 96-Well Thermal Cycler (Thermo Fisher Scientific), adding no-RT control of each sample. The quantitative RTPCR amplification was performed in a final volume of 10 ul with TaqMan Universal PCR Master Mix No AmpErase UNG (2x). The reaction mix was incubated 10 min at 95°C, followed by 40 cycles of 15 seconds at 95°C and 60 s at 60°C, in a LightCycler 96 instrument (Roche Applied Science), being the Ct values calculated using LightCycler software. All reactions were run as triplicates except the no-RT controls, the non-template controls, and the endogenous and exogenous controls that were run as duplicates. We considered a positive result when at least two out of the three replicates were positive, or at least three out of the six replicates were positive in the case of quantitative RTPCR repetition. Only the mean of the Cts with a standard deviation (SD) lower than 2 was included in the expression level analysis. When the SD was higher than 2 in the first quantitative RTPCR, we performed a second one, the mean of the Cts was included in the expression level analysis only if the SD of at least three replicates were lower than 2. The concentration of the miRNAs was quantified by normalizing with respect to a combination of the expression levels of the exogenous control: cel-miR-39 and the endogenous control: hsa-miR-126-3p. The ΔCt was estimated as previously detailed (21), HHV-6A/B miRNA normalized expression = 2^−^ΔCt = 2^−^[(HHV-6/B miRNA Ct)exogenous normalization - (endogenous miRNA Ct)exogenous normalization] = 2^−^[(HHV-6/B miRNA Ct - cel-mir-39-3p Ct) - (hsa-miR-126-3p miRNA Ct - cel-mir-39-3p Ct)].

### Anti-HHV-6A/B IgG and IgM Immunoassays

The anti-HHV-6A/B IgM titers in the serum samples of the patients were assessed using ELISA-VIDITEST anti-HHV-6A/B IgM kit (VIDIA), following the manufacturer's instructions. We considered negative samples those with an index value lower than 9, and positive samples with an index value higher than 11. Samples with index values between 9 and 11 were not included in the analysis of the prevalences.

The anti-HHV-6A/B IgG titers in both serum and CSF samples were assessed with ELISA-VIDITEST anti-HHV-6A/B IgG CSF (VIDIA), following the manufacturer's recommendations of the fabricant. Samples were equivocal when the range of concentration was between 10.5 and 14 AU/ml; in such cases, these samples were not included in the analysis of the prevalences. Samples whose concentration was lower than 10.5 AU/ml were considered negative, and the ones higher than 14 AU/ml were positive.

The HHV-6A/B antibody index (AI) values were calculated as previously described (22). Total IgG concentrations in serum and CSF were measured nephelometrically using an IMMAGE 800 (Beckman Coulter), and the total albumin with the AU5800 (Beckman Coulter). AI values higher than 2 were considered as indicative of intrathecal antibody production, lower than 1.5 of intrathecal antibody production not found, and values between 1.5 and 2 as suspect intrathecal antibody production, which were not included in the analysis of the prevalences.

### Clinical Data

The clinical variables considered for analysis were: MS disease duration (months), the time from the last relapse to the sample collection (months), Expanded Disability Status Scale (EDSS) score at sample collection, Multiple Sclerosis Severity Scores (MSSS) at the beginning of the treatment, number of relapses at sample collection, annual relapse rate from the beginning of the diseases until the onset of the treatment, and two radiological measurements: number of gadolinium (Gd) enhancing lesions and number of T2 lesions higher or lower of 10. Magnetic resonance imaging (MRI) of the brain was performed in 1.5 T scanners. Contiguous axial sections (slice thickness: 5 mm) were acquired to cover the entire brain. The sequences collected for this study were axial T2-weighted imaging, axial fluid-attenuated inversion recovery (FLAIR) T2, axial proton density T2-weighted imaging, and T1-weighted imaging with Gd-enhancement.

### Statistical Analysis

Numerical variables were expressed as mean ± standard deviation, or median (25th, 75th percentile), and categorical variables as percentages. Categorical variables were compared using the chi-squared test or Fisher's exact test, and numerical variables between groups using the Mann–Whitney U test. The nonparametric Spearman coefficient was applied to evaluate the correlation between two continuous variables. Subjects with missing data were omitted from the corresponding analyses. *P*-values <0.05 were referred to as statistically significant in the text. When necessary, the Bonferroni adjustment was performed, and the results were shown as *p*-value corrected (pcorr). All analyses were conducted using SPSS for Windows (Ver. 15.0) software (SPSS Inc.), and plots were elaborated with GraphPad Prism 5.01 and SPSS. The data that support the findings of this study are available from the corresponding author upon reasonable request.

## Results

One MS patient did not have enough CSF for performing HHV-6A/B miRNA analysis; therefore, only his serum sample was included in the study.

### Prevalences and Levels of HHV-6A/B miRNAs

Hhv6b-miR-Ro6-1 and −4 were not detected in serum or CSF samples of any patient. Regarding the ones that we detected (Table 2), the prevalences of hhv6b-miR-Ro6-2, −3-3p, −3-5p, and miR-U86 in both serum and CSF were not statistically different between MS and OND patients. Given the low prevalence of hhv6b-miR-Ro6-3-3p in both fluids, it was excluded from further statistical analysis. Similarly, the levels of hhv6b-miR-Ro6-2, −3-5p, and miR-U86 were not statistically different between MS and OND patients in both serum and CSF (data not shown).

**Table 2:** HHV-6A/B miRNAs prevalence in serum and cerebrospinal fluid (CSF) of multiple sclerosis (MS) and other neurological diseases (OND) patients.

	**hhv6b-miR-Ro6-2**		**hhv6b-miR-Ro6-3-3p**		**hhv6b-miR-Ro6-3-5p**		**miR-U86**	
	**Serum**	**CSF**	**Serum**	**CSF**	**Serum**	**CSF**	**Serum**	**CSF**
MS	97.7 (41/42)	81 (34/42)	4.8 (2/42)	2.4 (1/42)	95.2 (40/42)	50 (21/42)	54.8 (23/42)	11.9 (5/42)
OND	95.7 (22/23)	86.4 (19/22)	0	4.5 (1/2)	91.3 (21/23)	54.5 (12/22)	47.8 (11/23)	9.1 (2/22)

**Figure 1:**
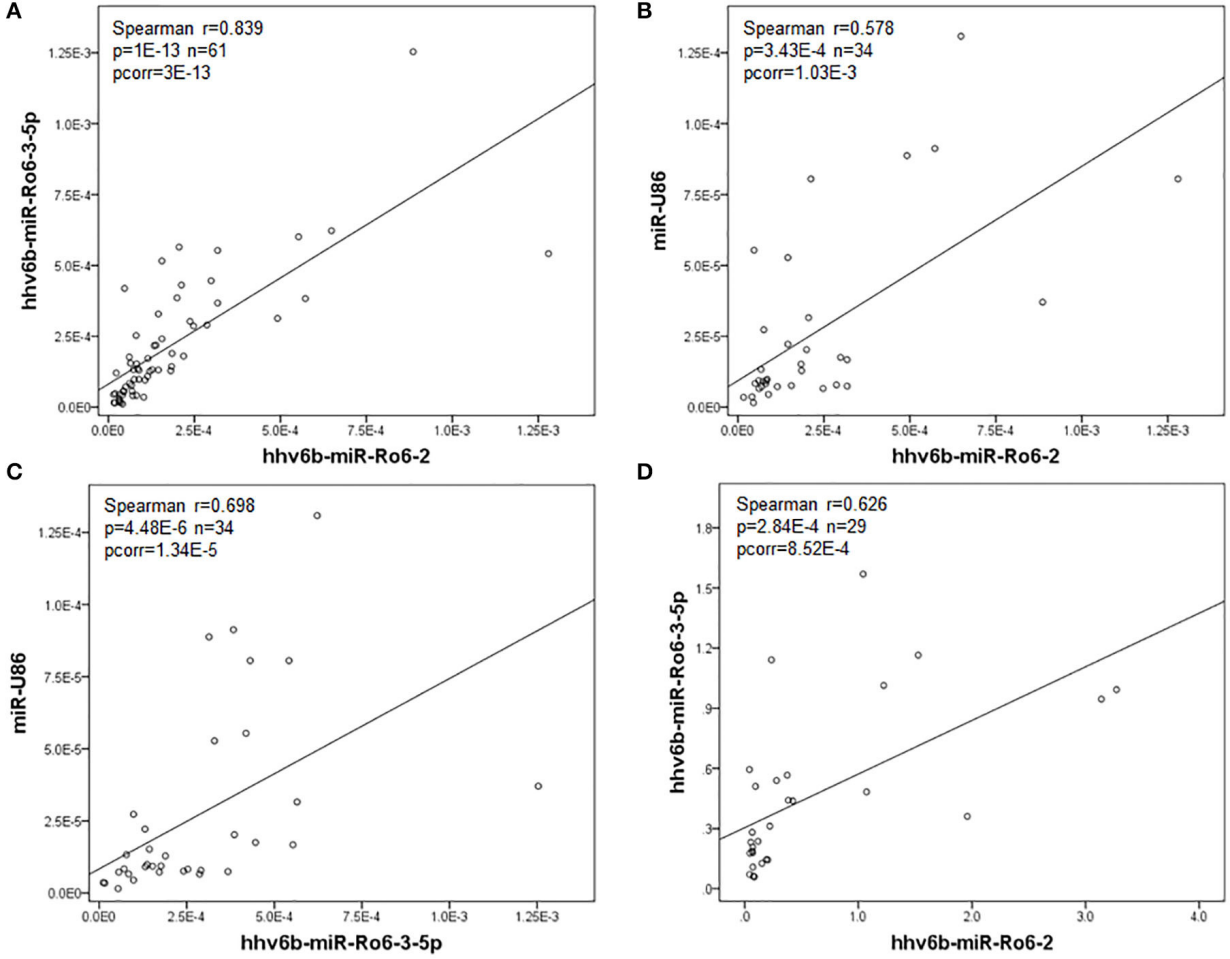
Correlation between the normalized expression levels of the HHV-6A/B miRNAs in serum **(A–C)** and cerebrospinal fluid (CSF) **(D)** in the whole population (multiple sclerosis and other neurological diseases patients). Pcorr, Bonferroni-corrected *p*-value.

In serum, we found a significant correlation between the levels of hhv6b-miR-Ro6-2, −3-5p, and miR-U86 in the whole population (MS and OND patients) (Figure 1), and also in both populations independently (Table 3). In CSF, we only found a correlation between hhv6b-miR-Ro6-2 and −3-5p (Figure 1, Table 3). Finally, there was no correlation between the levels of the analyzed miRNAs in serum vs. those in CSF, considering every miRNA (data not shown).

**Table 3:** Correlation of HHV-6A/B miRNAs levels between them in serum (left) and cerebrospinal fluid (CSF) (right) of multiple sclerosis and other neurological diseases patients.

**A. Multiple sclerosis patients**					
**SERUM**	**hhv6b-miR-Ro6-2**	**hhv6b-miR-Ro6-3-5p**	**CSF**	**hhv6b-miR-Ro6-2**	**hhv6b-miR-Ro6-3-5p**
**hhv6b-miR-Ro6-3-5p**	*r* = 0.823 *p* = 7.4E-11 pcorr = 2.22E-10 *n* = 40		**hhv6b-miR-Ro6-3-5p**	*r* = 0.529 *p* = 0.024 *n* = 18	
**miR-U86**	*r* = 0.434 *p* = 0.039 pcorr = 0.117 *n* = 23	*r* = 0.627 *p* = 0.001 pcorr = 0.003 *n* = 23	**miR-U86**	n/a *n* = 4	n/a *n* = 1
**B. Other neurological diseases patients**					
**Serum**	**hhv6b-miR-Ro6-2**	**hhv6b-miR-Ro6-3-5p**	**CSF**	**hhv6b-miR-Ro6-2**	**hhv6b-miR-Ro6-3-5p**
**hhv6b-miR-Ro6-3-5p**	*r* = 0.857 *p* = 7E-7 pcorr = 2.1E-6 *n* = 21		**hhv6b-miR-Ro6-3-5p**	*r* = 0.764 *p* = 0.006 *n* = 11	
**miR-U86**	*r* = 0.770 *p* = 0.006 pcorr = 0.018 *n* = 11	*r* = 0.793 *p* = 0.004 pcorr = 0.012 *n* = 11	**miR-U86**	n/a *n* = 2	n/a *n* = 1

*N, number of subjects; N/a, not applicable; r, Spearman's rank correlation coefficient; pcorr, Bonferroni-corrected p-value*.

### HHV-6A/B miRNAs in Serum Based on Anti-HHV-6A/B IgG and IgM Serology

The prevalence and titers of the anti-HHV-6A/B IgG in serum were significantly higher in the MS patients [100% (40/40), 51.5 (21.7, 120.4) AU/ml] than in the patients with OND [81% (18/22), 24.4, (14.6, 59.1) AU/ml] (*p* = 0.013, *p* = 0.006 respectively), but we did not find any difference for the anti-HHV-6A/B IgM. On the other hand, the mean value for the coefficient albumin concentration in CSF/albumin concentration in serum was 4.3 ± 1.9 (MS: 4.2 ± 1.5, OND: 4.5 ± 2.5).

**Figure 2:**
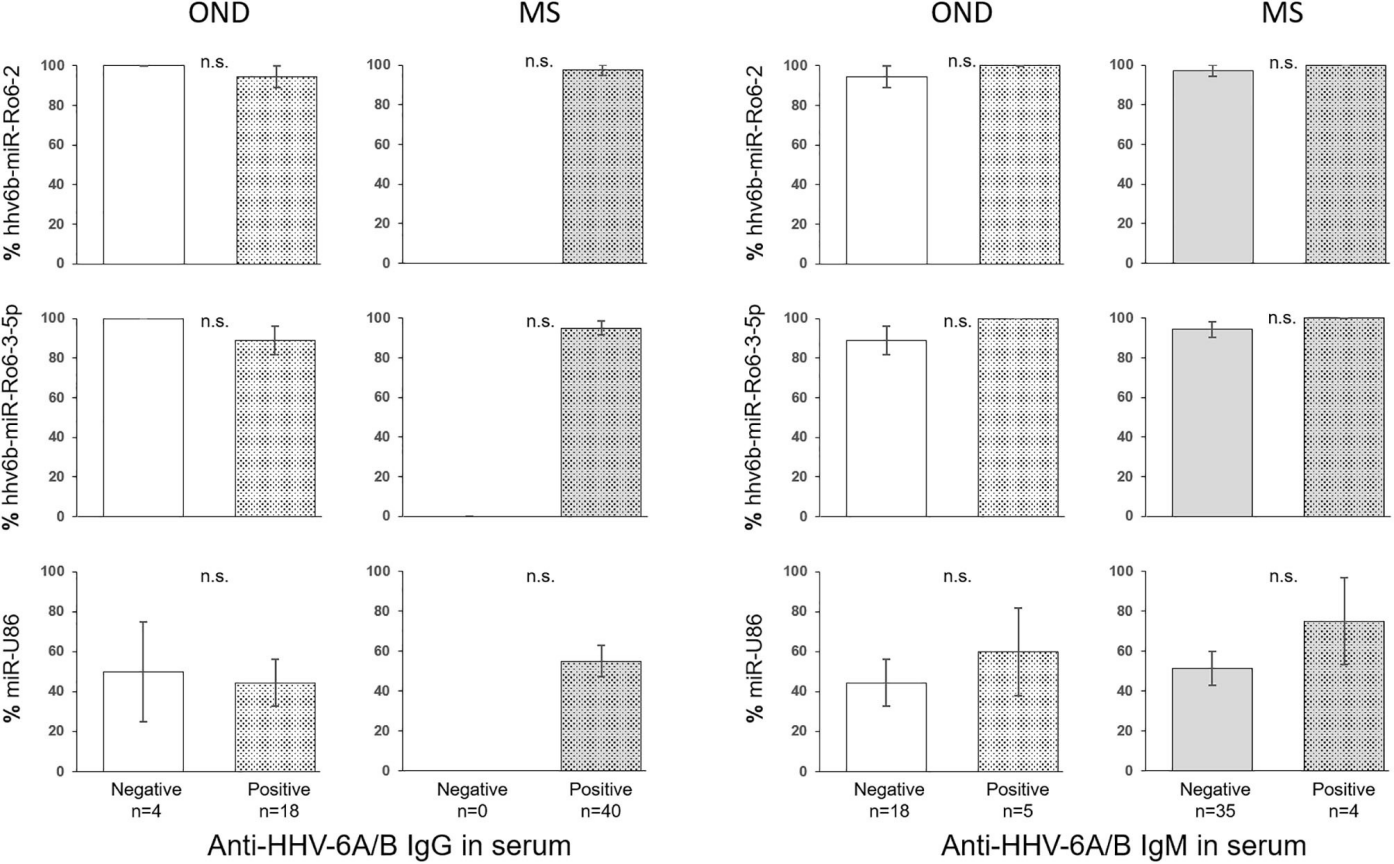
Prevalence of HHV-6A/B miRNAs in the serum of multiple sclerosis (MS) and other neurological diseases (OND) patients based on anti-HHV-6A/B IgG and IgM serology. The error bars depict the standard error of percentages.

**Figure 3:**
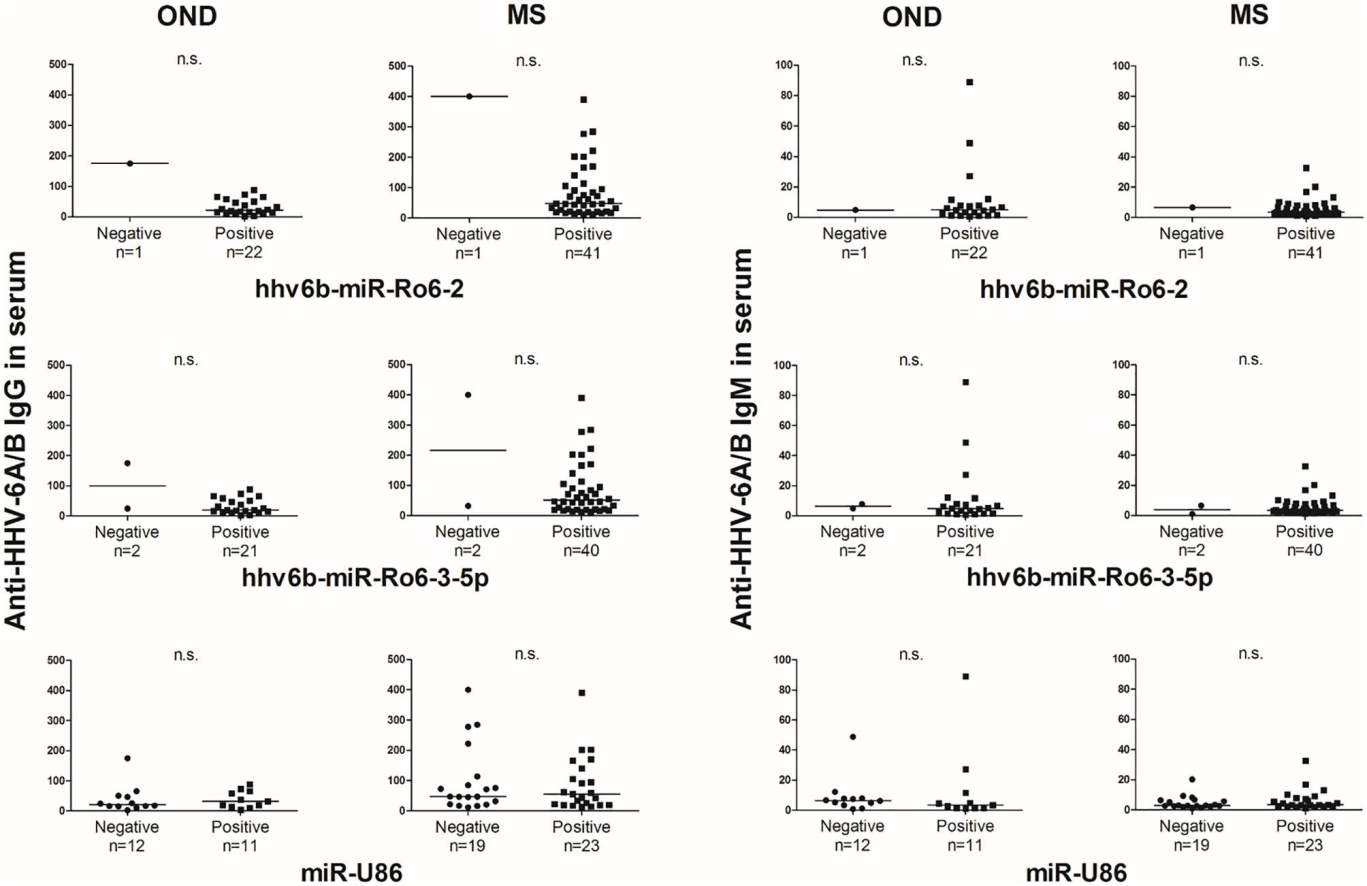
Titers of anti-HHV-6A/B IgG and IgM antibodies based on the presence of HHV-6A/B miRNAs in the serum of multiple sclerosis (MS) and other neurological diseases (OND) patients. Horizontal lines depict median values.

The prevalence of hhv6b-miR-Ro6-2, −3-5p, and miR-U86 in HHV-6A/B IgM or IgG positive and negative patients were not statistically different in OND, neither in MS patients (Figure 2). The titers of the IgG and IgM in serum between positive and negative miRNAs patients were similar in both MS and OND patients (Figure 3). We did not find any correlation between the levels of the miRNAs and the anti-HHV-6A/B IgG and IgM in serum (data not shown).

### HHV-6A/B miRNAs in CSF Based on HHV-6A/B IgG in CSF and Intrathecal Antibody Production

The anti-HHV-6A/B IgG prevalence and titers in CSF were not statistically different between MS [20% (8/40), 2.8 (0, 7.8) AU/ml] and OND [18.2% (4/22), 0.5 (0, 10.3) AU/ml] patients, neither the intrathecal HHV-6A/B antibody production [MS: 7.1% (3/42), 0.13 (0, 0.28); OND: 17.4% (23/19), 0.05 (0, 0.22)].

**Figure 4:**
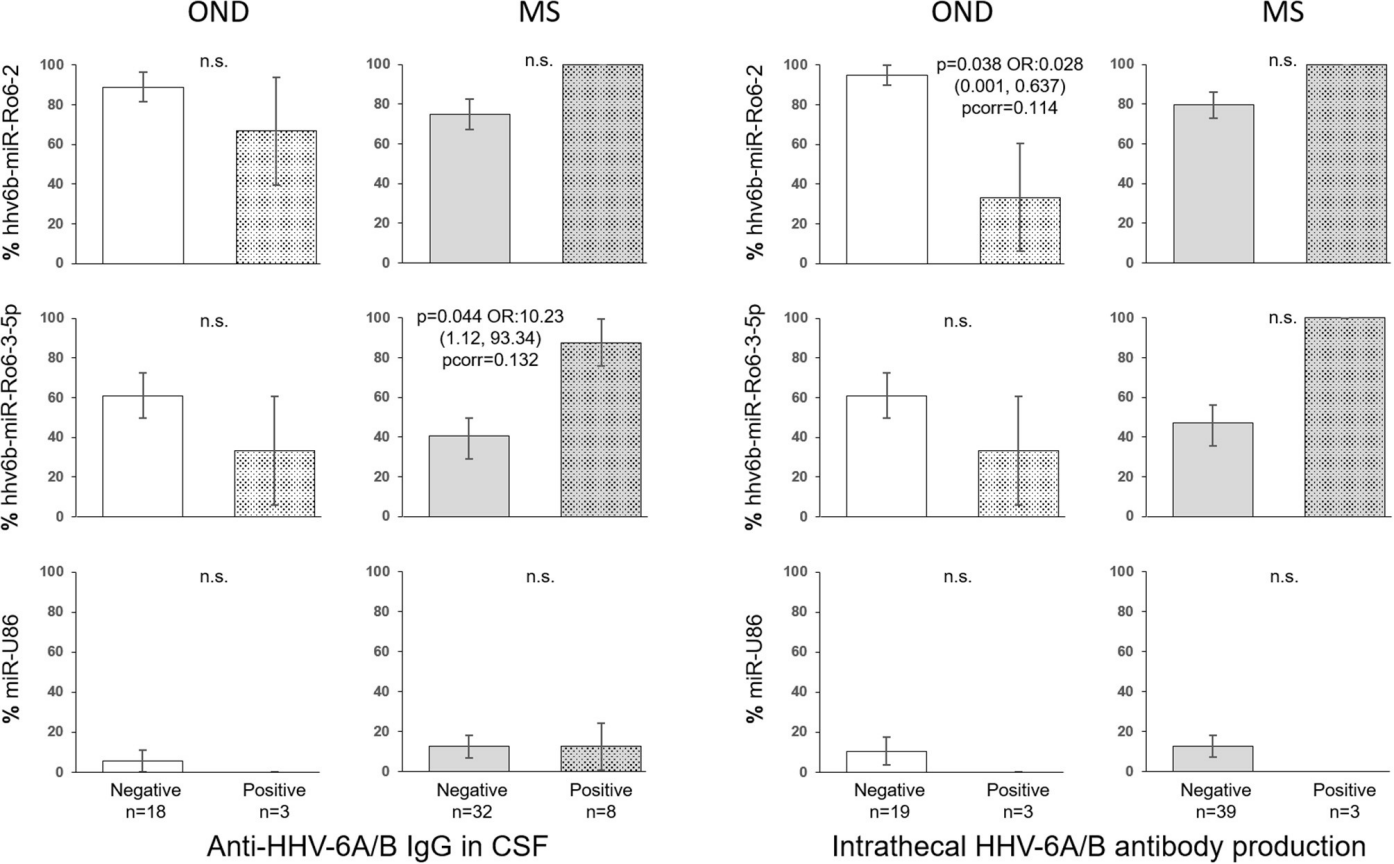
Prevalence of HHV-6A/B miRNAs in the cerebrospinal fluid (CSF) of multiple sclerosis (MS) and other neurological diseases (OND) patients based on intrathecal antibody production and anti-HHV-6A/B IgG in CSF. The error bars depict the standard error of percentages. Pcorr, Bonferroni-corrected *p*-value.

In the CSF of patients with OND, the prevalence of hhv6b-miR-Ro6-2 in the intrathecal HHV-6A/B antibody production positive patients was lower than in the negative ones (*p* = 0.038), although the *p*-value did not survive the Bonferroni adjustment (pcorr = 0.114) (Figure 4), the prevalence of hhv6b-miR-Ro6-3-5p went in the same direction. Similar results were obtained for hhv6b-miR-Ro6-2 and −3-5p prevalence based on anti-HHV-6A/B IgG in CSF. However, in MS patients, these trends for these miRNAs prevalence reversed. The most pronounced difference was for the prevalence of hhv6b-miR-Ro6-3-5p base on anti-HHV-6A/B IgG (*p* = 0.044), although the *p*-value did not withstand the Bonferroni correction (pcorr = 0.132).

**Figure 5:**
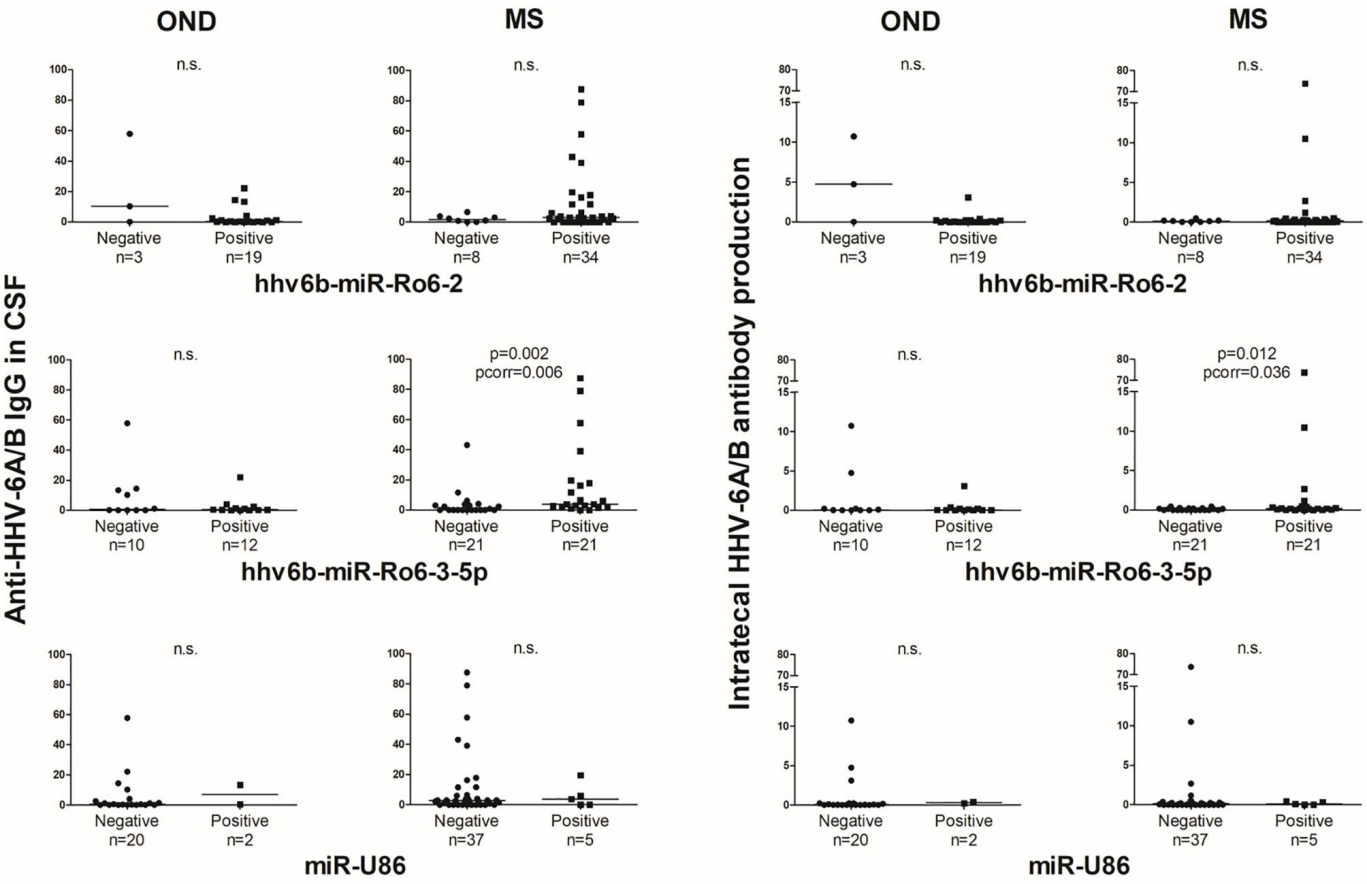
Titers of anti-HHV-6A/B IgG in CSF and intrathecal antibody production based on the presence of HHV-6A/B miRNAs in the cerebrospinal fluid (CSF) of multiple sclerosis (MS) and other neurological diseases (OND) patients. Horizontal lines depict median values. Pcorr, Bonferroni-corrected *p*-value.

The anti-HHV-6A/B IgG levels in CSF and the intrathecal antibody production based on the presence of hhv6b-miR-Ro6-2 and −3-5p showed a similar trend that the prevalence results (Figure 5). However, it is in MS patients, where we found statistically significant differences.

### Association Between HHV-6A/B miRNAs and Clinical Variables

The intrathecal antibody production was positively associated with the number of relapses before the sample extraction (*p* = 0.017 *r* = 0.367) in MS patients.

We did not find any association between the analyzed miRNAs in serum and the selected clinical parameters in the whole population of MS patients. However, in CSF, we found an association between miR-U86 in CSF and Gd-enhancing lesions. We did not detect the miR-U86 in the 15 MS patients with active lesions in MRI, whereas we detected it in 30.8% (4/13) of the patients without active lesions (*p* = 0.035), although the *p*-value did not withstand the Bonferroni correction (pcorr = 0.105). Additionally, the number of these lesions was statistically higher in the miR-U86 negative patients [1 (0, 3)] than in the positive ones [0, (0, 0), *p* = 0.036, pcorr = 0.108]. However, we did not find any association between the presence or levels of hhv6b-miR-Ro6-2, −3-5p, or miR-U86 and having more or <10 T2 lesions.

## Discussion

We did not detect hhv6b-miR-Ro6-1 and −4 in any subject of the study, MS or OND patient, neither in serum nor CSF. Likewise, the prevalence of hhv6b-miR-Ro6-3-3p was very low in both groups of patients. Miyashita et al. (23) studied the levels of HHV-6B miRNAs and HHV-6B DNA in patients with DIHS/DRESS (drug-induced hypersensitivity syndrome/drug reaction with eosinophilia and systemic symptoms), and obtained similar results to ours regarding the practically undetectable levels of hhv6b-miR-Ro6-1, −3-3p and −4.

MiRNAs of both HHV-6B (16) and −6A (17) were identified infecting various T lymphocyte cell lines (Sup-T-1 for HHV-6B, and HSB2 and Jjhan cells for HHV-6A) with different strains of these viruses (Z29 and HST for HHV-6B, and U1102 for HHV-6A). Therefore, the authors suggested that these miRNAs derived from the viruses during lytic infection, without discarding that they could be expressed in latently infected cells as well as in chromosomally integrated HHV-6A/B patients. The high prevalence of hhv6b-miR-Ro6-2 and −3-5p, especially in serum, found in our study would indicate their potential role in the maintenance of the latency state. Hhv6b-miR-Ro6-2 lies antisense to the predicted B2 (ORF); meanwhile, hhv6b-miRro6-3-5p lies antisense to B1 ORF. The IE genes B1 and B2 are exclusive of HHV-6B and, although the function of these genes is unclear, it is believed that they are important to initiate viral infection (6). Therefore, we speculate that these two miRNAs would downregulate the expression of these two genes, favoring the HHV-6B latency instead of its reactivation. Virally encoded miRNAs of other herpesviruses have also been shown to promote latency through targeting viral transcripts, such us several miRNAs of the human simplex virus (HSV-1) (24, 25), of the human cytomegalovirus (HCMV) (26), of the EBV (27) and those of the Kaposi's sarcoma-associated herpesvirus (KSHV) (28).

Nukui et al. (17) identified miRNA-U86 from the HHV-6A following a very similar experimental approach to that of Tuddenham et al. (16) previously mentioned. This miRNA inhibits the expression of HHV-6A IE2 protein; what is more, the deletion of miRNA-U86 promotes viral DNA accumulation, which would suggest that this miRNA control HHV-6A lytic replication. When Tuddenham et al. identified the HHV-6B miRNAs, they claimed that these miRNAs were conserved in HHV-6A. However, Nukui et al. did not detect them. The correlation between the miRNAs in serum could be explained by the fact that these miRNAs have a similar potential function in the maintenance of the latency state of the two viruses that, although different, are very close to each other. On the other hand, it is suggested that HHV-6B is more seroprevalent than HHV-6A, which could explain that the prevalence of the miRNA-U86 is considerably lower than those of hhv6b-miRro6-2 and −3-5p, in both serum and CSF.

In serum, we detected a higher anti-HHV-6A/B IgG prevalence and titers in MS patients compared to OND patients. Most previous studies found this difference between MS patients and controls (28, 29). However, in OND, even the patients without detectable HHV-6A/B IgG levels had a very high prevalence of HHV-6A/B miRNAs, indicating that these patients had already been in contact with the virus. After our results, it is likely that the ratio of infection to be higher than 95% (30). The prevalence of the intrathecal antibody production close to 10% was slightly inferior to the 20% described in previous publications (31, 32). This intrathecal HHV-6A/B antibody production is unknown, whereas it could be due to an HHV-6A/B reactivation, or part of a polyspecific B cell activation (3). Maybe the understanding of the HHV-6A/B miRNAs role could help to clarify this point. No association between HHV-6A/B miRNAs and anti-HHV-6A/B antibodies in serum was found in MS or OND patients. However, we found an association between the antibodies in the CSF and the prevalence of hhv6b-miR-Ro6-2/-3-5p that was inversed depending on the patients, MS or OND. CSF reflects what is happening in the brain, given that the BBB is separating CNS from the systemic immune system, maybe it is why we found the association only in this fluid. In these patients, the coefficient between albumin concentration in CSF and serum were in all cases higher than 9, indicating that the blood-brain barrier (BBB) remains intact.

OND patients without anti-HHV-6A/B IgG in CSF or absence of intrathecal antibody synthesis showed a trend to have a higher prevalence of hhv6b-miR-Ro6-2 and −3-5p than positive patients. We presume that an increase of the humoral immune reactivity to HHV-6A/B reflects a more acute infection and/or reactivation. Therefore, these results support the hypothesis of a possible role of these two miRNAs in the maintenance of the latency state. This trend was lost in MS patients, even reversed. This could be the consequence of the deregulation of the immune system characteristic of these patients, even for control viral infection, as it has been previously suggested (3). Some of these trends were statistically significant, but none of them withstand the Bonferroni correction; therefore, these results have to be interpreted with caution. These inversed trends between MS and OND were also found for the anti-HHV-6A/B IgG levels and the intrathecal antibody production based on the presence of hhv6b-miR-Ro6-2 and −3-5p; where the MS patients with detectable hhv6b-miR-Ro6-3-5p in CSF had statistically significant higher levels of anti-HHV-6A/B IgG and intrathecal antibody production than those without this miRNA.

Finally, miR-U86 seemed to follow different patterns from hhv6b-miR-Ro6-2 and −3-5p. The researchers who identified it (17) claimed that this miRNA was exclusive of the HHV-6A. The inability of our ELISA to distinguish between anti-HHV-6A or−6B antibodies could hinder finding an association of this miRNA with the antibody produced exclusively against it. Recently, it has been described a novel multiplex serological assay measuring IgG reactivity that can distinguish between HHV-6A and −6B (33). The use of this assay and detecting the two viral DNA would clarify the expected association between the analyzed miRNAs and the anti-HHV-6A/B antibodies in further studies.

Regarding the results related to clinical variables, we described a mild positive correlation between the intrathecal antibody production and the number of relapses before the sample extraction. These results would agree with previous studies (34, 35) that establish a relationship between high anti-HHV-6A/B IgG and IgM titers and risk of relapse. Besides, and despite our low number of patients, we found an inversed association between miR-U86 in CSF and the presence of Gd-enhancing lesions. With the other analyzed miRNAs, both in CSF or serum, we did not find any association with the selected clinical variables.

Previous articles (12, 36, 37) showed a higher presence of HHV-6A/B DNA in patients with contrast-enhancing lesions, indicating a higher prevalence of HHV-6A/B in active MS plaques. In the CSF of our MS patients with Gd-enhancing lesions in MRI, we did not detect miR-U86, whereas we detected it in 30% of the patients without active lesions, and the number of these lesions was higher in the miR-U86 negative patients than in the positive ones. Although these results are trends, since the *p*-values did not survive the Bonferroni correction, we cautiously interpreted them as Nukui et al. proposed, the inhibitory role of miR-U86 over the protein U86 would help to maintain the latency state, hindering the HHV-6A reactivation and the brake of the BBB and consequently the presence of the Gd-enhancing lesions (38). Nevertheless, our hypothesis disagrees with that of Prusty et al. (39).

For the first time in the literature, we detected the miRNAs from the HHV-6A and −6B in MS patients. The high prevalences of hhv6b-miR-Ro6-2 and −3-5p could suggest a possible role in the maintenance of the latency state, Further studies are warranted not only to validate these results but also to try to understand the HHV-6A/B life cycle and consider these miRNAs as possible biomarkers of the pathologies related to this virus.

## Data Availability Statement

The raw data supporting the conclusions of this article will be made available by the authors, without undue reservation.

## Ethics Statement

The studies involving human participants were reviewed and approved by Ethics Committee of the Hospital Clínico San Carlos. The participants provided their written informed consent to participate in this study.

## Author Contributions

AN-G, MG-M, and SP-P performed the experiments and updated the database with the clinical and experimental data. MD-M, RA, and RA-L conceived the study, analyzed the data, and drafted the manuscript. All authors contributed to the article and approved the submitted version.

## Conflict of Interest

The authors declare that the research was conducted in the absence of any commercial or financial relationships that could be construed as a potential conflict of interest.
